# Electrocardiographic approach strategies in patients with Parkinson disease treated with deep brain stimulation

**DOI:** 10.3389/fcvm.2024.1265089

**Published:** 2024-04-12

**Authors:** Carlos Rafael Sierra-Fernández, Luis Rodrigo Garnica-Geronimo, Alejandra Huipe-Dimas, Jorge A. Ortega-Hernandez, María Alejandra Ruiz-Mafud, Amin Cervantes-Arriaga, Ana Jimena Hernández-Medrano, Mayela Rodríguez-Violante

**Affiliations:** ^1^ Department of Medical Education, National Institute of Cardiology Ignacio Chávez, Mexico, Mexico; ^2^ Department of Movement Disorders, National Institute of Neurology and Neurosurgery Manuel Velasco Suárez, Mexico, Mexico

**Keywords:** electrocardiography, electrophysiology, frequency filters, artifact, deep brain stimulation, movement disorders, Parkinson’s disease, neurostimulation

## Abstract

Deep brain stimulation (DBS) is an interdisciplinary and reversible therapy that uses high-frequency electrical stimulation to correct aberrant neural pathways in motor and cognitive neurological disorders. However, the high frequency of the waves used in DBS can interfere with electrical recording devices (e.g., electrocardiogram, electroencephalogram, cardiac monitor), creating artifacts that hinder their interpretation. The compatibility of DBS with these devices varies and depends on factors such as the underlying disease and the configuration of the neurostimulator. In emergencies where obtaining an electrocardiogram is crucial, the need for more consensus on reducing electrical artifacts in patients with DBS becomes a significant challenge. Various strategies have been proposed to attenuate the artifact generated by DBS, such as changing the DBS configuration from monopolar to bipolar, temporarily deactivating DBS during electrocardiographic recording, applying frequency filters both lower and higher than those used by DBS, and using non-standard leads. However, the inexperience of medical personnel, variability in DBS models, or the lack of a controller at the time of approach limit the application of these strategies. Current evidence on their reproducibility and efficacy is limited. Due to the growing elderly population and the rising utilization of DBS, it is imperative to create electrocardiographic methods that are easily accessible and reproducible for general physicians and emergency services.

## Introduction

Deep brain stimulation (DBS) is an interdisciplinary and reversible therapy that delivers high-frequency electrical stimulation to specific brain sites through implanted electrodes to correct aberrant neural pathways underlying motor and cognitive neurological disorders (1).

Although initially used for the treatment of essential tremor and Parkinson's disease (PD), the use of DBS has expanded to the treatment of other refractory movement disorders (dystonia, tics) (2–4), psychiatric disorders (major depressive disorder, obsessive-compulsive disorder, addictions) (5–7), and more recently, other neurodegenerative diseases such as Alzheimer's disease (8).

The high-frequency waves used in neurostimulation range from 100 to 200 Hz, which can interfere with electrical recording devices (e.g., electrocardiogram, electroencephalogram, cardiac monitors), creating artifacts that limit or impede their interpretation. The variability in compatibility between DBS and these devices depends on both modifiable and non-modifiable factors of both systems (9, 10). However, there is no consensus on strategies to reduce the electrical artifacts created by DBS in patients with movement disorders, which becomes particularly relevant in emergencies where the acquisition of an electrocardiogram (ECG) is imperative.

An increasingly aging population, the growing number of people with movement disorders, and the increased use of DBS will inevitably lead primary care physicians to encounter patients with some form of neurostimulation. Given the lack of knowledge about DBS among the non-specialized medical community and the absence of protocols for its electrocardiographic approach, this review provides a compilation of its functioning, the electrocardiographic artifacts it produces, and the existing strategies to reduce them.

### Epidemiology

#### Parkinson's disease and deep brain stimulation statistics

Neurodegenerative diseases are the leading cause of disability-adjusted life years and the second leading cause of mortality worldwide (11, 12). PD is the second most common neurodegenerative disease globally, second only to Alzheimer's disease, affecting 2%–3% of the population over 65 years old (13). The worldwide prevalence of PD is estimated to be between 100 and 300 per 100,000 individuals (14), and in Mexico, this disease affects 1.6%–2.3% of the population over 65 years old, a figure similar to that reported in the rest of Latin America (15). The incidence of PD has dramatically increased in the past three decades (16, 17). From 1990 to 2016, the number of people worldwide with PD doubled to 6 million, a number projected to double again by the year 2040 (17, 18), making PD the fastest-growing neurological disorder in the world (16, 19), a phenomenon that some authors have referred to as the Parkinson's pandemic. Although the cause of PD cannot be attributed in 90% of cases (20), increased longevity in the population and expanded diagnostic strategies partially explain this growth phenomenon. Genetic predisposition and environmental exposure contribute significantly to the increasing global incidence (21), estimated to be 108–202 per 100,000 individuals over 65 years old in the United States (22) and approximately 10.2 per 100,000 individuals in Mexico (23). Incidence and prevalence are 1.5–2 times higher in men (14).

Only 2% of eligible PD patients are estimated to receive DBS treatment (24, 25), indicating that DBS is an underutilized therapeutic option.

National registries on DBS placement are limited; however, as of 2021, it was estimated that over 200,000 patients with movement disorders had been treated with DBS devices worldwide, which continues to increase each year (26).

#### Cardiovascular diseases in patients with Parkinson's disease

Cardiovascular diseases represent one of the leading causes of morbidity and mortality in patients with PD (27–29). The association between PD and acute myocardial infarction has been inconsistent, with some studies demonstrating that individuals with PD have a significantly higher risk of developing acute myocardial infarction than the general population (30–33). In contrast, others have not reported significant differences (34). Both conditions share pathophysiological processes and an age-related increase in incidence (31, 33). On the other hand, some cardiovascular risk factors have even been identified as protective against developing PD (35–38).

Other diseases, such as heart failure, autonomic dysfunction, arrhythmias, and conduction disorders, are particularly prevalent in individuals with PD (39–44). Some reports have linked PD to prolonged QT interval and the development of malignant arrhythmias, likely secondary to autonomic dysfunction and pharmacological treatment, closely related to the risk of sudden death, estimated to occur in 3%–4% of PD patients (45–48).

Diabetes mellitus and systemic arterial hypertension are particularly prevalent comorbidities in individuals with PD (30, 49, 50). Studies conducted in Mexico highlight a high prevalence of cardiometabolic diseases and elevated cardiovascular risk among patients with PD (51, 52). Furthermore, individuals with diabetes mellitus have up to a 23% higher likelihood of developing PD (53).

#### Cardiovascular morbidity and mortality in deep brain stimulation patients

Cardiometabolic diseases prevail among individuals with movement disorders receiving DBS treatment (54). Studies that have retrospectively analyzed the main reasons for emergency department visits in DBS patients have focused solely on those related to the neurostimulation device itself (55–57). However, it has been recognized that age and comorbidities, particularly coronary artery disease, directly influence readmission rates and hospitalization time in these patients (58, 59). The mortality rate in PD patients treated with DBS is 8.2%–21.4% at five years (60–62) and 23%–30.4% at ten years (54, 62), with acute myocardial infarction being the second or third leading cause of death in these patients (54, 60, 63).

### Deep brain stimulation

#### Fundamental concepts

Neurostimulation is the nervous system's electrical, selective, and reversible modulation through invasive and noninvasive techniques. There are different types of neurostimulation based on the neuroanatomical area they modulate. DBS is characterized by delivering electrical stimuli to the basal ganglia. These stimuli are generated by an implantable pulse generator (IPG) and delivered through extension wires connected to electrodes (64). Most IPGs have a lithium battery with a lifespan of 7–10 years. However, recently rechargeable systems and even those powered by sources such as thoracic movement during the respiratory cycle have been implemented (65). These devices are coated with titanium and are subcutaneously implanted, usually in the infraclavicular area. The placement of electrodes in the exact neuroanatomical area is done through stereotactic surgery, and each electrode is constructed of platinum-iridium, which provides excellent conductivity and minimal toxicity (66).

On the other hand, the extension wires are subcutaneously placed in the lateral portion of the neck and are made of a nickel alloy covered with a polyurethane sheath (66). At their ends, each electrode has one or more contacts from which the impulses emerge outward, and the spatial arrangement of these contacts determines the shape and extent of the generated electric field (66, 67). The electrical impulses are short (60–450 μs), have a frequency ranging from 100 to 200 Hz, and amplitude between 2.0–5.0 mV (67, 68), parameters comparable to those used in conventional pacemakers. DBS placement is generally bilateral, although, in some patients, unilateral neurostimulation is sufficient (67, 68).

DBS models exhibit heterogeneity, yet they share common characteristics that allow for modification in configuration or deactivation through an external controller. The two principal configurations of neurostimulation, monopolar and bipolar, govern the flow of electrical current within the implanted electrode and the implanted pulse generator (IPG). In the monopolar configuration, the current emanates from one or several contacts of the implanted electrode (cathode) to the IPG (anode), generating a broader electromagnetic field (EF). In contrast, bipolar stimulation involves the simultaneous activation of two electrode contacts, with one serving as the anode and the other as the cathode. This configuration confines the electrical current to a more localized area.

The distinction between bipolar and monopolar configurations extends to their impact on the Volume of Tissue Activated (VTA). Computational modeling indicates distinct spatial distributions of stimulation effects. Monopolar configurations tend to produce a more extensive VTA, whereas bipolar configurations, activating two electrode contacts concurrently, result in a more focused and intricate pattern of neural modulation.

Furthermore, it is important to note that DBS leads can function interchangeably as cathodes or anodes, providing versatility in the modulation of neural activity. This flexibility extends to the choice between voltage-based and current-based stimulation. In voltage-based stimulation, the device sets a specific power level, allowing for adaptability but requiring careful consideration of individual variability. On the other hand, current-based stimulation maintains a consistent current output, ensuring a more predictable and controlled delivery of electrical stimulation (69). The versatile selection in stimulation configuration enriches the adaptability of DBS interventions, enabling tailored approaches that align with patient-specific needs and response profiles. This flexibility empowers customized strategies in DBS interventions, catering to the unique requirements and responses of individual patients (69–72).

#### Mechanism of action and anatomical areas

The effects of DBS are immediate, reversible, and pleiotropic, as they depend on factors specific to the electrical system of the IPG and the intrinsic characteristics of the stimulated tissue (e.g., types of ion channels, myelin content, orientation of nerve fibers), which means that the effects on one specific brain area may not apply to another (1). Although the exact DBS mechanism of action remains unclear, in the context of PD various theories have been proposed, including the prevention and modification of abnormal neural pathways' propagation, neurochemical modification of synapses, and the neuronal microenvironment (1, 73–77). The beneficial effect of DBS is likely a combination of several elements.

The control of voluntary movements is a process that occurs at cortical and subcortical levels, where the basal ganglia play an essential role in the initiation and cognitive modulation, dependent on the balance between glutamatergic excitatory pathways and GABAergic inhibitory pathways (78–82). This balance is disrupted in movement disorders. In PD, the loss of dopamine in the substantia nigra to the striatum causes overactivation of the direct excitatory pathway through the internal globus pallidus and inhibition of the indirect path through the external globus pallidus and the subthalamic nucleus, resulting in a more significant inhibition of the thalamus and its cortical inputs, leading to the akinesia and rigidity characteristic of PD (67, 82–84). DBS therapy aims to silence the pathologically hyperactive pathways through internal globus pallidus or subthalamic nucleus stimulation (68). Different therapeutic targets are preferred for treating other movement disorders, such as the ventral intermediate nucleus of the thalamus for essential tremor, the most common movement disorder in the world (84).

#### Indications, contraindications, and adverse effects

Although initially conceived as a panacea for movement disorders, the most significant benefit of DBS appears reserved for individuals with a specific phenotype of PD (Table 1). While there is no defined age range, DBS is recommended for patients younger than 70 years (85–87). Generally, monopolar stimulation is preferred over bipolar stimulation, as it requires a lower stimulation intensity to achieve the same clinical effects. This is often attributed to the more diffuse electric field in monopolar setups. The broader distribution of current allows for a greater volume of neural tissue to be influenced, requiring less current density at any given point to produce the desired effects (66). Nevertheless, the choice between monopolar and bipolar stimulation isn't solely determined by the required stimulation intensity. Side-effect thresholds, referring to the point at which unwanted effects or discomfort occur, differ between the two montages. While the more focused electric field in bipolar setups may have a higher risk of localized side effects due to the higher current density, this could also mean a more precise and controlled modulation of the desired area (66).

**Table 1:** Indications and contraindications for deep brain stimulation (DBS) placement in patients with Parkinson's disease (PD) (64, 86).

Indications	Contraindications
Adequate response to levodopa dopaminergic treatment	Patients unable to operate the neurostimulator properly
Refractory dyskinesia	Secondary parkinsonism
Refractory fluctuations	Severe cerebral atrophy
Absence or mild cognitive impairment	Basal ganglia vascular involvement
Absence or reasonable control of psychiatric illness	Uncontrolled psychiatric disorders, particularly major depressive disorder

The presence of a cardiac pacemaker had previously been considered a contraindication for DBS implantation (85, 88). However, some case series have reported that the coexistence of both devices in the same patient does not pose a greater risk, with the bipolar configuration being preferred over the monopolar to avoid interference (88, 89). The main adverse effects of DBS include dysarthria, balance impairment, visual disturbances, tonic contractions, and behavioral changes such as mania, apathy, and severe depression (67, 90). Considering these adverse effects, the variability in response, and the inherent risks of surgical intervention, DBS is reserved for patients with a specific profile of PD.

### Deep brain stimulation as an electrocardiographic artifact

**Figure 1:**
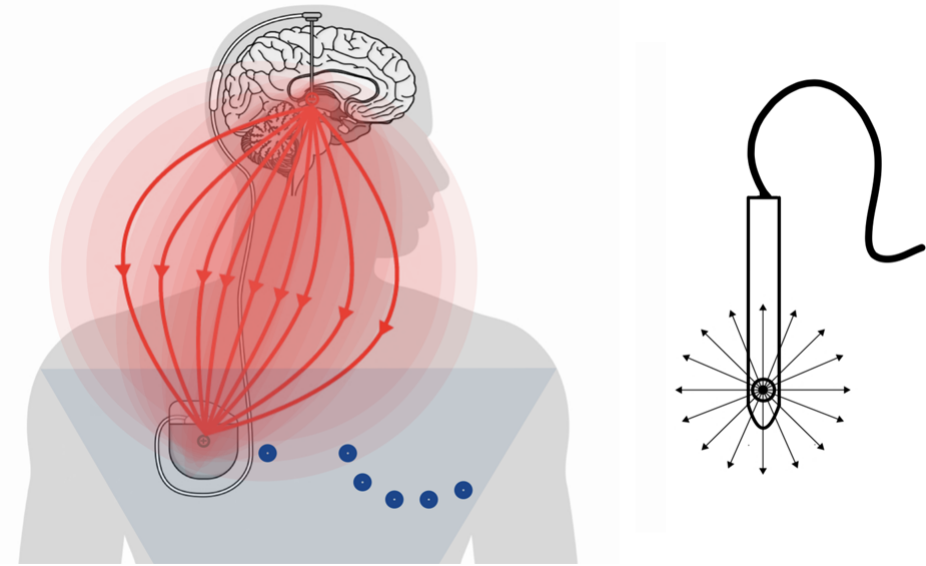
Left image: in the monopolar configuration, the generated electric field exhibits a greater extension and reaches the precordium, overlapping with the recording area of the electrocardiogram (arrangement of leads I, II, and III is represented by Einthoven's triangle). Right image: The electrode generates an eccentric electric field, from the cathode to the anode.

The VTA and the extent of the EF generated during neurostimulation are crucial when considering DBS as a potential artifact for other electrical recording devices. In the monopolar configuration, the EF generated spans an area from the implanted electrode to the IPG, reaching the precordial region, creating electrical artifacts and interference with other stimulation and recording devices located in the same anatomical area (e.g., pacemakers, ECG, cardiac monitors) (9, 10) (Figure 1).

**Figure 2:**
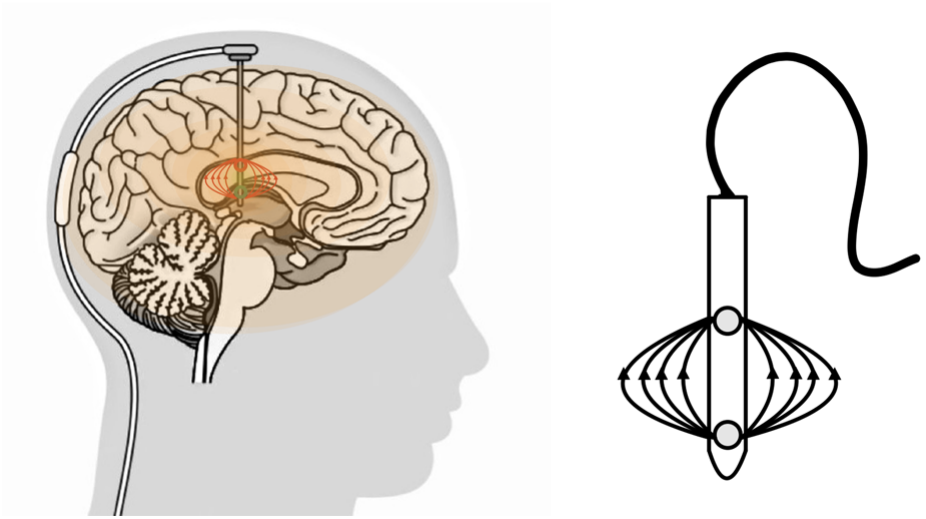
Left image: in the bipolar configuration, the electric field is confined to a more circumscribed area and rarely reaches the precordium. Right image: There is simultaneous activation of two electrode contacts, with one serving as an anode and the other as a cathode.

It should be noted that vectors from the electrical artifact resulting from monopolar stimulation can be observed continuously throughout the body's electric field. This is consistent with the more diffuse electric field generated by monopolar setups, where the current flows from a single electrode to a reference point. Moreover, the generation of an observable artifact by monopolar stimulation can be attributed to the distinct voltage decay characteristics between monopolar and bipolar configurations (91). Maxwell's equations describe the fundamental principles governing electromagnetic fields, providing insights into how electric fields propagate and decay in different configurations (92). However, in a bipolar configuration, the EF is more circumscribed and rarely reaches the precordial area, generating significantly fewer artifacts and interference (93) (Figure 2).

Artifact resulting from a bipolar montage typically resides below the noise floor in peripheral recording electrodes, such as EKG leads. This implies that the artifact generated by bipolar stimulation is less prominent or more challenging to detect in recordings from peripheral electrodes. The specific spatial characteristics of the bipolar configuration might contribute to a reduced impact on distant recording sites (94).

As mentioned before, Maxwell's equations provide a mathematical description of how the electric field produced by different electrode configurations (e.g., monopolar, or bipolar) will propagate through the body but also can be applied to analyze the voltage decay (how the electric field strength diminishes with distance from the stimulation source). This provides a theoretical foundation for understanding the generation, propagation, and decay of electric fields, and their principles are essential for comprehending the biophysics of electrical stimulation and the resulting artifacts in the body's electric field. Applying these principles allows researchers and clinicians to optimize stimulation parameters and electrode configurations for specific applications.

Other factors directly influencing the quality of recordings are the stimulation voltage and the underlying disease (93). The infraclavicular position, whether left or right, does not show differences in artifact generation (93).

**Figure 3:**
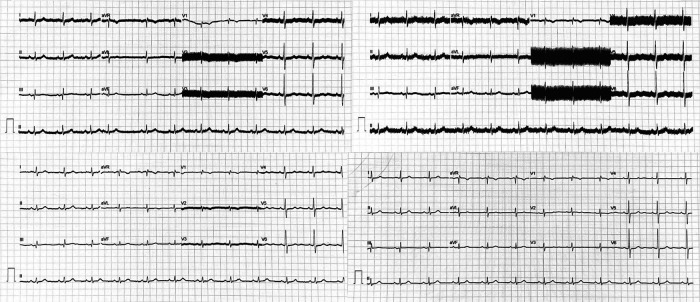
Twelve-lead electrocardiograms in a patient with deep brain stimulation (DBS) in monopolar configuration at 159 Hz: Top left image: monopolar at 100 Hz, an artifact affecting all leads is observable, with V2-V4 showing more pronounced effects. Top right image: monopolar with a 150 Hz filter, as the filter is close to the DBS stimulation frequency, the artifact becomes markedly more conspicuous. Bottom left image: Monopolar with a 20 Hz filter, significant reduction of artifact is noted, albeit slightly discernible in V2 and V3. Bottom right image: Switch to the bipolar configuration at 100 Hz, complete disappearance of the DBS artifact is achieved, rendering the electrocardiogram indistinguishable from that of an individual without DBS. ECG: Electrocardiogram, DBS: Deep Brain Stimulation.

Under standard settings, modern electrocardiograms can record frequencies between 0.05–150 Hz, including the impulses generated by DBS (100–200 Hz). Capturing these frequencies during an ECG or long-term recordings (e.g., Holter monitor) can generate artifacts during the recording, mimicking abnormal rhythms (e.g., atrial fibrillation, atrial flutter) and even making interpretation impossible (9, 68, 93–98) (Figure 3). This problem becomes particularly relevant in a cardiovascular emergency, where obtaining an ECG is imperative.

Multiple authors have emphasized the importance of enhancing the medical community's knowledge in managing DBS, addressing the challenges involved in its handling, and establishing a protocol for electrocardiographic approaches (10, 93, 99, 100).

### Current strategies for electrocardiographic approach in patients with deep brain stimulation

Several studies and case reports have demonstrated the effectiveness of various electrocardiographic strategies to improve the quality of electrocardiographic recordings in the presence of artifacts caused by deep brain stimulation (DBS).

I. Changing the DBS configuration

For the reasons described above, switching the device to a bipolar configuration significantly reduces the artifact produced by monopolar DBS, allowing for interpretable electrocardiographic recordings in most cases (68, 93).

II. Temporarily deactivating DBS during electrocardiographic recording

Temporary deactivation of DBS is the definitive intervention to eliminate the electrocardiographic artifact. This approach can be done using the device controller or by placing a magnet over the generator pulse implant for <5 s. Deactivating one of the devices can significantly attenuate the artifact when the patient has bilateral stimulation (68). However, this strategy does not appear to be superior to changing the configuration, as total deactivation, albeit momentary, results in the return of abnormal movements, leading to motion artifacts in the ECG. The resting tremor frequency in Parkinson's disease is 4–6 Hz (100), which can mimic abnormal rhythms and limit interpretability (10, 101–103.) In a study of 100 patients with Parkinson's disease, Hwang et al. described the presence of various electrocardiographic artifacts, with 78% showing baseline variations and 11% exhibiting abnormal rhythms similar to atrial flutter/fibrillation (104). Different techniques have been described to attenuate this artifact in patients with movement disorders, such as electrode placement on limb roots (99). However, further studies are needed to evaluate their effectiveness during electrocardiographic recording in patients with DBS.

III. Application of frequency filters

**Figure 4:**
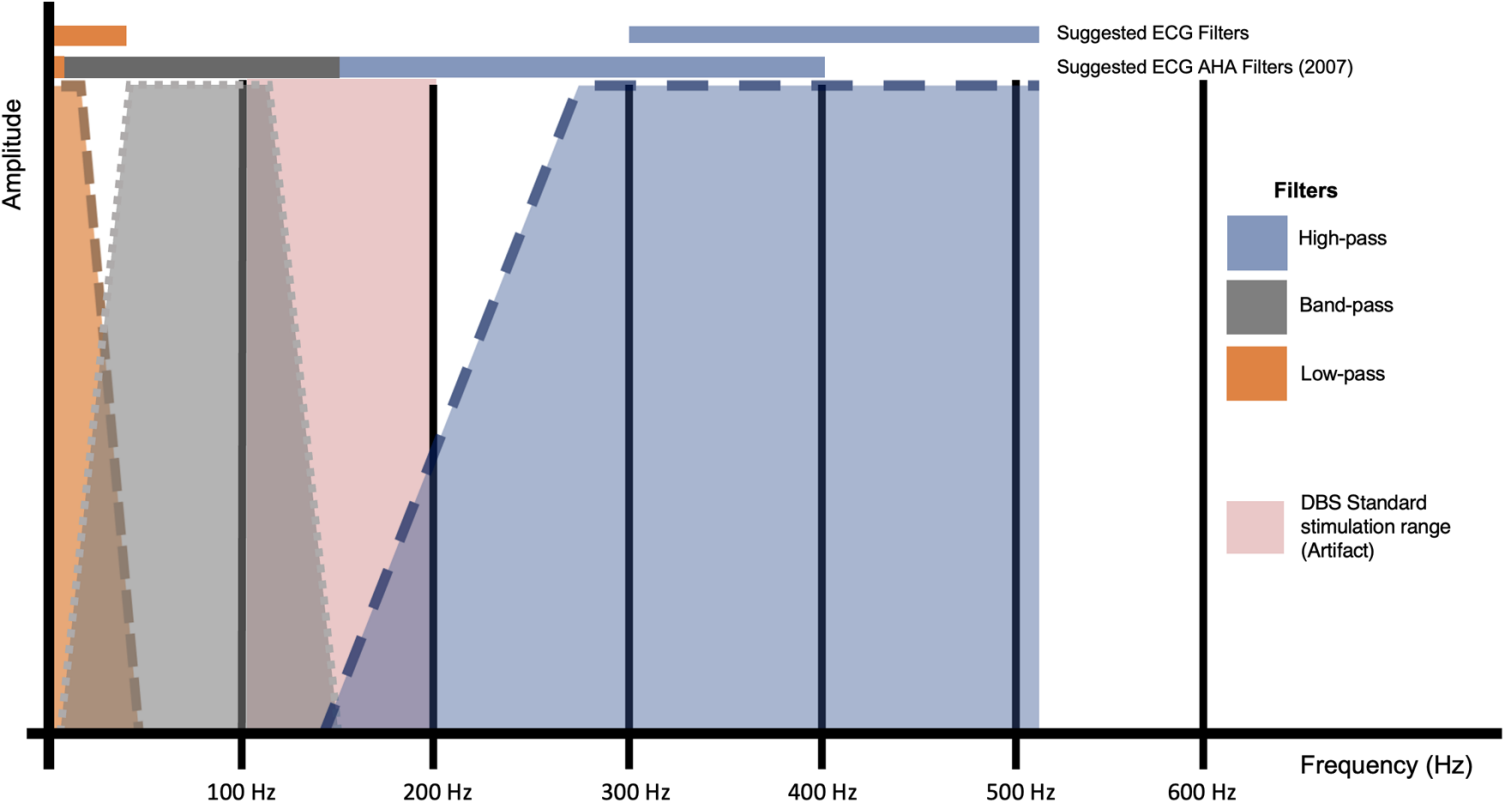
Comparative analysis of suggested filters and filters standardized by the American heart association (AHA) (105) in a bode diagram on their effect on the detection of heart electrical activity concerning the artifact generated by the standard stimulation frequency used in DBS treatment for Parkinson's disease (100-200 Hz).

EKG machines incorporate robust filtering mechanisms to isolate and record the electrical activity of the heart. These filters typically include low-pass filters to eliminate high-frequency noise and high-pass filters to attenuate low-frequency interference. Traditional EKG devices allow operators to manually adjust filters based on specific needs. However, it's important to note that some EKG models have fixed filters that cannot be manually changed. The unique challenges posed by DBS artifacts require a specialized approach beyond standard recommendations by organizations like the American Heart Association (AHA) (105). Recent studies highlight effective cutoff frequencies for DBS artifact reduction. For instance, implementing a low-frequency cutoff filter around 40 Hz has proven effective in mitigating artifacts related to the average frequency of DBS pulses (100–200 Hz) (68). Additionally, applying filters above the DBS pulse range (300–512 Hz) has shown significant artifact reduction (98) (Figure 4). A cutoff filter that attenuates artifacts from abnormal movements has not been determined.

IV. Use of non-standard leads for electrocardiographic recording

**Figure 5:**
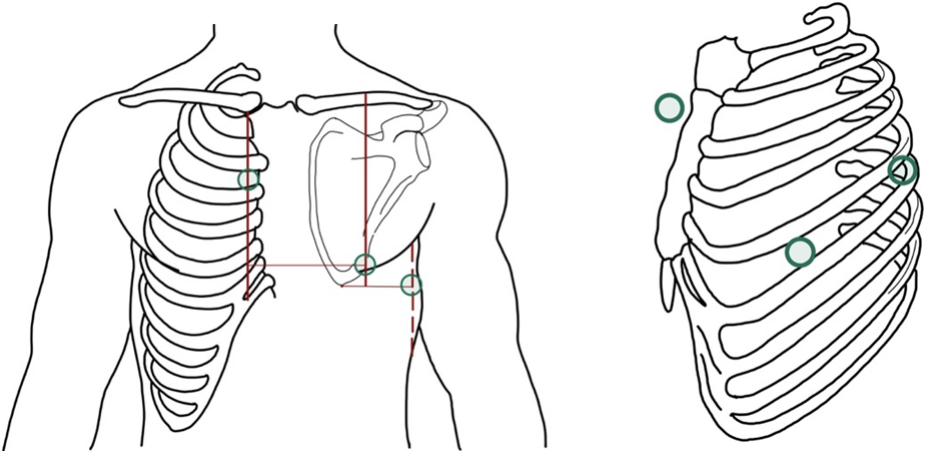
Nehb-Spöri leads, with limb leads (R.A., LA, and L.L.) placed on the right parasternal line at the level of the second intercostal space (A), left axillary posterior line at the level of the scapular apex (D), and left midclavicular line at the fifth intercostal space (J), respectively, thus reducing artifact caused by distal movement in Parkinson's disease after deactivation of deep brain stimulation (DBS). Parasternal Line (PSL), intercostal space (ICS), Left Axillary Posterior line (LAP), Left midclavicular line (LMC).

Using alternative leads to the classic 12 leads described by Wilson can help capture cardiac vectors without overlapping with the EF of monopolar DBS configuration. Mruk et al. demonstrated that using Nehb-Spöri leads (Figure 5) significantly reduces the artifact caused by the return of abnormal movements after deactivating monopolar DBS (94), likely attributed to the exclusion of peripheral leads, which are more susceptible to motion artifacts. A reported case by Steltzer et al. described a significant reduction of DBS artifacts when using devices with less than 12 leads (KardiaMobile 6l, Apple Watch) (98). It is possible that other non-standard leads (e.g., right-sided, posterior, Lewis, Medrano leads) could improve the quality of electrocardiographic recordings in these patients.

## Discussion

### Main limitations of current strategies

Although effective, these approaches are not always reproducible. Changing the polarity and deactivating DBS are strategies that require medical personnel who are familiar with manipulating neurostimulation devices. Modifying the neurostimulator is not applicable in all contexts, whether due to lack of experience or unavailability of the controller at the time of medical intervention. Variability among DBS models can affect the operator's ability to deactivate or modify the device's configuration.

While the application of frequency filters to eliminate artifacts induced by DBS has demonstrated effectiveness in specific case reports, there is a dearth of evidence comparing it to alternative strategies, and its efficacy compared to different DBS configurations still needs to be verified. Furthermore, the ability to manually manipulate filters in traditional EKG equipment confers a level of adaptability that proves invaluable in the landscape of electrocardiographic approach in DBS patients. In contrast, EKG models with fixed filters mandate adherence to predetermined settings, emphasizing the critical importance of judicious equipment selection for DBS studies. Filtering capabilities wield direct influence over the accuracy with which neural signals are captured and analyzed.

While electrocardiographic recordings with non-standard leads provide an alternative perspective to the classic 12 leads, they limit the comprehensive electrocardiographic analysis of the heart and the application of standardized measures for detecting atrial and ventricular enlargements.

Some case reports have highlighted the challenges in managing DBS devices during cardiovascular emergencies, where obtaining an electrocardiogram is imperative, leading to delays in diagnosis and treatment (93, 99).

It is generally recommended that patients undergoing DBS treatment have an electrocardiogram and cardiovascular assessment as future reference (89).

### Potential developments in the field

Portable cardiac monitoring devices (e.g., smartwatches) can identify abnormal rhythms and perform single-lead electrocardiographic recordings, which have shown effectiveness in patients with DBS (96). These observations encourage the implementation of such devices in patients receiving neurostimulation.

Although a cutoff filter to reduce abnormal movement artifacts has not been determined, recent technological advances have enabled the development of signal-filtering techniques for the electrical signal of the heart. These techniques have successfully reduced various types of noise in electrocardiograms, such as electromyographic noise. This progress allows for the acquisition of cleaner and clearer electrocardiographic signals, facilitating their accurate interpretation (106).

Recently, programming modalities for DBS have been investigated to allow neurologists or neurosurgeons to make remote and real-time adjustments to the neurostimulator's configuration, enabling necessary modifications when electrocardiographic assessment is required (105, 107). Additionally, some designs of electrodes with multiple contacts have demonstrated improved control over the VTA and generated EF New designs of miniature DBS generators would allow placement in the patient's skull, limiting EF projection towards the precordium during monopolar DBS activation (108, 109).

## Conclusions

Current strategies, although effective, are not replicable in all contexts. This issue, raised in an increasingly aging society where DBS is a widely used therapy, calls for electrocardiographic approach strategies in these patients that are affordable and reproducible for primary care physicians and emergency services.
